# Color without light

**DOI:** 10.1017/S1478951525100862

**Published:** 2025-10-01

**Authors:** Henry Bair

**Affiliations:** Wills Eye Hospital, Philadelphia, PA, USA

The first time a patient tried to tell me what color felt like without sight, I realized how little of the world I had been noticing.

He was in his seventies, a former farmer with hands that seemed permanently stained by earth. Advanced glaucoma had taken nearly everything – faces at arm’s length, street signs, and the small ciphers of daily life. He could navigate by sound and habit and the stubborn hope that this visit might yield some trick we had not tried.

I checked intraocular pressures and pupil reactivity, looked at an optic cup that had enlarged. I said the words I am trained to say – about eye drops and adherence, about nerve fibers and visual fields. He nodded eagerly as though my routine counsel carried the power to change everything.

“What do you miss?” I asked, because the room was quiet and my next patient had yet to show up.

“Tomatoes on the vine,” he said without hesitation. “Not the look. The way the afternoon presses on your face while you pick them. The smell when the stem snaps, like a rubber band.” He added, “And snow. A whole morning becomes one piece. The sound gets padded. The air against your teeth is colder than the air against your cheek. You know before you open the door.”

He was telling me about color, but he never said a color. He spoke in temperatures, weights, sounds, odors – stacking sensations in place of what light used to do. I had the urge to translate, to supply labels he avoided, but it felt like walking into someone else’s house without knocking.

At the door he said, “I still see colors. Not with my eyes. With the places they’ve been. You know?”

I did not, not really.

For days I carried his words. On a Saturday between call shifts, I visited the nearby Philadelphia Museum of Art because I had a few hours that did not belong to duty or sleep. Near the information desk was a small sign I had never spotted, offering audio guides for visitors who are blind or partially sighted. I borrowed a device and let a stranger’s voice guide me.

The first stop was Van Gogh’s *Sunflowers.* The voice did not begin with color. “A tall vase sits firmly on a narrow table, bursting with sunflowers that push outward in every direction. The petals are thick and curling, some soft as felt, others dry and brittle. The flower centers are rough and seedy like coarse sandpaper. Stems jut at angles, stiff and knobby, crowding the vase so tightly you can almost hear them creak.”

In the modern galleries I encountered one of Mark Rothko’s monumental paintings. The voice: “Let your weight settle. The canvas is taller than you, unframed edges breathing a little into the room. Two rectangles hover one above the other: the upper block is dense and even, its edges softened as if smudged with the side of a thumb; the lower block is shorter and looser, worked wet, the brush leaving wide, wavering strokes you could almost count. If you dragged a fingertip over it, you would feel a rhythm of little hills.” I closed my eyes and traced the air in front of me with my hand. The voice went on. “What you’re looking at is not a picture of anything but a set of physical cues – absorbent cloth, scumbled strokes, a breathing edge – teaching your body how to sense stillness.”

Nearby, a small marble statue by Constantin Brancuși carried a label I could not stop thinking about: *Sculpture for the Blind*. It felt like permission, a reminder that art had always been more than what the eye alone could hold.

The audio guide’s descriptions did not pretend I could not see; it simply did not rely on sight to do the job. It gave me temperature, heft, and motion – a way to look with parts of myself that were not tied to my eyes. I began to understand that color, for my patient, had been converted into a set of coordinates that memory and body could map even when light could not: the tongue’s tally of spices, the skin’s register of warmth and pressure, the ear’s catalog of echoes.

I thought of my parlance in clinic. “Your nerve looks pale,” I had said to a hundred people, untroubled by the fact that half the room might not use that adjective in the way I had just used it. “Your conjunctiva is white and quiet.” “Your cornea is clear.” The literature of my training came preloaded with sighted metaphors. It had never felt like an exclusion until it did.

Several months later, I saw the carpenter again.

“Any changes?” I asked.

“Nothing new,” he said, “but I did manage to find my blue shirt.” He grinned. “You know, the one that fits like a hug.”

I nearly repeated the color, then stopped. “The one with the heavier fabric?” I said instead. “The cuff that slides less on your wrist?”

“That’s the one.”

We went through the ritual: the adjustable chair, the chin on the slit lamp, the light he still disliked but endured. Before I leaned in with the scope, I said, “I’m going to bring the light closer. If it’s too much, just tell me ‘back up.’ If you need quiet, say ‘pause.’ I know I forget sometimes.”

He nodded. “I’ll just say ‘enough’ if I need a break.”

The examination gave us what it always gives: measurements, snapshots of a process that does not reverse. But something else changed. I narrated differently, not to perform or fill space, but to offer the kind of coordinates he had given me.

“The nerve we always talk about,” I said, “where all the wires gather before they leave the eye – the nerve has a center hollow we call the cup. You can think of it like the furrow left in a field. A little furrow is normal. But when water keeps running through, the groove gets deeper and eats into the rows around it. That’s what pressure does to the cup. It digs until the surrounding rim wears away.”

We adjusted his drops. We talked about labeling his kitchen, about marking the first and last notch on the stove with a raised dot so a burner could be set by counting. I printed a referral for low-vision services and said aloud what that meant: people who would measure his life, not just his eyes, and help redesign the parts that could be redesigned.

At the end of the visit he said, “You sound different today.”

“I listened to an art tour,” I said, feeling a little foolish for a moment. “It taught me how to see without color.”

He thought for a moment, then nodded. “My mother had this connection with the world back at the farm,” he said. “She could tell rain was coming, not by the what the sky or clouds looked like, but by how the air pressed on her skin. She knew when the soil was ready for planting from the way it clumped in her palm, not from its color.”

For a moment, the exam room became a classroom, and I the student.

Our profession trains us to be objective: to measure and compare, to strip away subjectivity. That discipline is useful and essential – but it also narrows what we notice. It leaves little room for describing how patients continue to sense the world, even when vision is gone.

Patients remind us that there are other ways of seeing. They widen the frame, showing that attention itself can be trained. You can learn to hear when a patient’s account is so much more than the chart in front of you, to sense when silence speaks louder than facts. You can describe a painting not by its colors but by its textures and weight, or a bowl not by its hue but by where its surfaces meet and break. In medicine, as in art, we can build encounters that do not rely only on what our eyes report, but on the wider field of human perception.

I do not romanticize loss. The clinic is full of people who would trade my vocabulary for one extra line of visual acuity. But between loss and nothing is a lot of ground. On that ground, naming matters. The right names let people orient, decide, and prepare. The wrong ones leave them feeling like the only reality is what they can no longer do.

The next day in clinic, another patient asked what would happen if his disease took the rest of his already-limited sight. We talked a little about low-vision services and tools – but mostly we explored together how a day could still be navigated without seeing. Onions are browned when the sizzle softens and sweetness rises; shoes can be matched by the feel of stitching under a finger, subway trains announce themselves by the screeches echoing through the tunnels, the pitch dropping just before the doors part and, the draft at your calves.

It was not a cure, but we were making a map together.

I still see color when I look at a painting or a nerve or the sky through a clinic window at the hour when the day softens its grip. I do not know what my patients see in those moments, not from inside. But I am learning to ask better questions: How does your kitchen tell you when it is morning? Where in your day does the air change temperature? What places in your week are loud with one kind of sound and quiet with another?

Light left. Color stayed. It moved into touch and taste and other small, delicate instruments of the body. My part is to pay attention and to speak in that language without assuming it is a deficit. It is, simply, another way the world announces itself.

